# Synthesis and Characterization of Chitosan-Decorated Nanoemulsion Gel of 5-Fluorouracil for Topical Delivery

**DOI:** 10.3390/gels8070412

**Published:** 2022-06-30

**Authors:** Asif Nawaz, Muhammad Shahid Latif, Maha Abdallah Alnuwaiser, Shafi Ullah, Muhammad Iqbal, Mulham Alfatama, Vuanghao Lim

**Affiliations:** 1 Advanced Drug Delivery Lab, Gomal Centre of Pharmaceutical Sciences, Faculty of Pharmacy, Gomal Univesity, Dera Ismail Khan 29050, Pakistan; asifnawaz676@gmail.com (A.N.); shahidlatif1710@gmail.com (M.S.L.); shafikustian@gmail.com (S.U.); iqbalmiani@gmail.com (M.I.); 2 Department of Chemistry, College of Science, Princess Nourah bint Abdulrahman University, P.O. Box 84428, Riyadh 11671, Saudi Arabia; maalnoussier@pnu.edu.sa; 3 Faculty of Pharmacy, Universiti Sultan Zainal Abidin, Besut Campus, Besut 22200, Terengganu, Malaysia; mulham4122@yahoo.com; 4 Advanced Medical and Dental Institute, Universiti Sains Malaysia, Bertam, Kepala Batas 13200, Penang, Malaysia

**Keywords:** 5-fluorouracil, chitosan, nanoemulsion, gel, topical delivery

## Abstract

(1) Background: The present study aimed to prepare chitosan-coated nanoemulsion gel containing 5-fluorouracil for enhanced topical delivery. (2) Methods: To formulate the nanoemulsion gel, oleic acid was used as the oil phase and Carbopol 940 as a gelling agent. Chitosan was used as a coating agent to control the release of 5-FU. Drug–excipient compatibility was evaluated using ATR-FTIR. The prepared nanoemulsion formulations were characterized based on particle size distribution, zeta potential, % encapsulation efficiency and drug content. In vitro drug release, skin drug retention and ex vivo permeation profiles were performed across rat skin using a Franz diffusion cell. Skin irritation experiments were also conducted on rats to examine the irritation potential of the formulations. (3) Results: It was found that the drug and excipients were compatible and chitosan successfully coated 5-FU, as demonstrated by ATR-FTIR results. The introduction of chitosan increased the size and zeta potential of the nanoemulsion. The 5-FU release in vitro was significantly lowered in the case of chitosan-decorated nanoemulsion (5-FU-C-NE), whereas the permeation and skin drug retention were higher in the case of 5-FU-C-NE. The formulations were proven non-irritant to the skin of the rats. The optimized formulation of the nanoemulsion was introduced into 1% Carbopol 940 gel. Incorporating the nanoemulsion into the gel further reduced the drug release in vitro and ex vivo permeation, whereas the retention of the drug in the skin was significantly increased (ANOVA; *p* < 0.05). The increase in skin retention was due to the presence of chitosan and Carbopol 940. The in vitro and ex vivo results were also confirmed with in vivo studies. Incorporating nanoemulsion into gel has resulted in higher T_max_, longer half-life and greater skin drug retention. (4) Conclusion: The results suggest that chitosan-decorated nanoemulsion gel is safe and can potentially be used to promote 5-FU skin retention, which is ideal for skin diseases such as melanoma.

## 1. Introduction

The use of topical application of nanoemulsion gels has recently been increasing. Drug delivery into the body via contact with the skin is considered a topical drug delivery [1]. Skin is regarded as one of the major barriers to entry of chemicals/drugs due to its complex structure. The stratum corneum, the outermost layer of skin, is the main challenge and barrier to the percutaneous absorption of topically applied drugs [2].

Different skin diseases, such as basal cell carcinoma, malignant lesions and premalignant cutaneous lesions, are often found in the deeper layers of skin. Therefore, the maximum amount of drug retention in the lower layer of the skin is required to treat these skin diseases [3]. In managing topical lesions such as skin cancers and actinic keratosis, 5-fluorouracil (5-FU) is widely used [4]. One of the major problems of 5-FU is its poor permeability preventing the achievement of the therapeutic concentration via conventional dosage forms such as solutions, ointments or creams [5]. Various techniques/approaches have been suggested to enhance the transdermal delivery of the hydrophilic drugs, i.e., 5-FU [6]. One of the approaches is the use of nanocarriers. Nanotechnology has become a vast field for biomedical applications, and nanocarriers such as polymeric nanoparticles, micelles, dendrimers, solid lipid nanoparticles, quantum dots and magnetic nanoparticles are widely studied [7]. Many studies have been conducted on 5-FU-loaded nanocarriers such as liposomes, solid lipid nanoparticles, ethosomes, niosomes and nanoemulsions. A nanoemulsion is a colloidal carrier system containing water, oil and emulsifying agent (surfactant) [8].

Nanoemulsions have high kinetic stability, small droplet size, low optical transparency and viscosity, making their use helpful for managing various dermatological diseases [9]. Their creaming, sedimentation and separation are prevented by high kinetic stability and a lower droplet size [10]. The significant effect of topical systems, enhanced drug solubility and good stability are the properties of utmost importance in the pharmaceutical industry [11]. The release, permeation and stability at the nanoscale are the properties of nanoemulsion that can easily penetrate via numerous skin barriers. The anatomical illness, patient preference, type of active ingredients and physicochemical features of the delivery system mainly affect the penetration of drug across the skin [12]. The coating/decoration of nanoemulsion with polymers such as chitosan is widely used technique because of its high-efficiency [13]. Chitosan is a polyelectrolyte, a biodegradable and biocompatible positively charged polymer used as a controlling agent in the drug delivery [14]. Chitosan can also be used as a thickener to stabilize nanoemulsions. The purpose of coating the drug/nanoemulsion droplet is to reduce the drug’s premature release and enhance permeation and skin drug retention. Previously, chitosan-modified nanocarriers of 5-FU were also developed and studied for their in vitro release and anticancer activities [15,16].

The nanoemulsion possesses low viscosity that restricts its use in managing various skin diseases [17]. Polymers employed in the formulation of gels have weak interactions with surfactants and the modified rheological behavior of nanoemulsions has been explored [18]. For transdermal application, Carbopol 940 was used to enhance the viscosity of the formulated nanoemulsion [19]. Adding a gel matrix to the nanoemulsion converts it into a nanoemulgel. The nanoemulgel was found to be more helpful for transdermal applications.

The present study aimed to formulate chitosan-decorated nanoemulsion gel containing 5-FU for deeper penetration into skin cancers through a topical route. The prepared formulations were characterized and investigated in vitro and ex vivo conditions and for skin drug retention.

## 2. Results and Discussion

### 2.1. Evaluation of 5-FU–Excipients Interactions (by ATR-FTIR)

**Figure 1:**
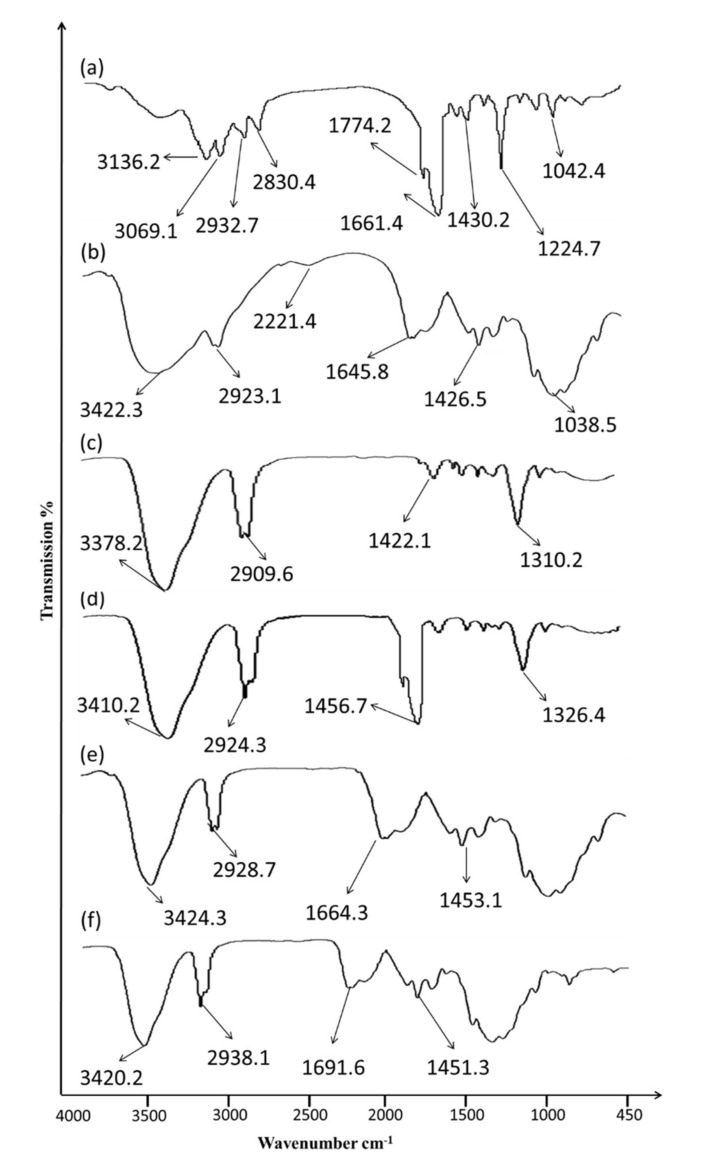
ATR−FTIR spectra of (**a**) 5-FU, (**b**) chitosan, (**c**) blank nanoemulsion (**d**) 5-FU-NE, (**e**) 5-FU-C-NE and (**f**) 5-FU-C-NE-G.

Pure 5–FU, chitosan, nanoemulsion (5-FU-NE, 5-FU-C-NE) and nanoemulsion gel (5-FU-C-NE-G) were subjected to ATR-FTIR as demonstrated in Figure 1. 5-FU depicted characteristic stretching bands at 3136.2 cm^−1^ (—NH) and symmetric aromatic ring stretching at 1774.2 cm^−1^ (C=O), 1661.4 cm^−1^ (C—N), 1224.7 cm^−1^ (C—F) and 1042.4 cm^−1^ (C—O). Chitosan characteristics peaks were obtained at 3422.3 cm^−1^ (—OH stretching), 2923.1 cm^−1^ (—CH_2_ asymmetric stretching), 1645.8 cm^−1^ (—CO) and 1426.5 cm^−1^ (C—O—C). The spectrum of 5-FU-NE exhibits strong absorption bands at 3410.2, 2924.3, 1456.7, and 1326.4 cm^−1^ that ensure the entrapment of 5-FU in the nanoemulsions. However, the distinctive peak of 5-FU in the 5-FU-NE was not observed. The surface of the nanoemulsion was evaluated by means of ATR-FTIR spectroscopy. With 5-FU-NE, the drug entrapped inside 5-FU-NE and the quantity of 5-FU is inconsequential for detection in contrast to 5-FU-NE. However, a minute amount of 5-FU might be observed on the surface of the nanoemulsion. The ATR-FTIR study depicted the absence of chemical interactions between 5-FU and excipients used in the nanoemulsion formulation [20]. This indicates that 5-FU primarily resides inside the core of the NE. The Carbopol gel spectra showed one OH stretching peak at 2938.1 cm^−1^, a prominent C—O peak at 1691.6 cm^−1^ and peaks at 1451.3 cm^−1^ attributable to the C—O and O—H functional groups. The 5-FU characteristics peaks were not identified in the nanoemulsion gel spectra, demonstrating entrapment/encapsulation of 5-FU in chitosan and Carbopol layers.

### 2.2. Physicochemical Characterization of Nanoemulsion

#### 2.2.1. Size, Zeta Potential and Surface Morphology

The addition of chitosan to the nanoemulsion increased droplet size significantly (*t*-test, *p* < 0.05) (Table 1). The droplet size of the nanoemulsion was in the range of 92.1–119.3 nm. The droplets depicted for the prepared formulations were in the nanometer range and a low value for the polydispersity index was observed. The reduced droplet size is of utmost importance in nanoemulsion because it offers greater surface area and results in better drug partition and absorption. In the literature, the exact consensus regarding droplet size of nanoemulsions is not mentioned [21].

The prepared formulations exhibited low polydispersity values, which showed uniformity of droplets in formulations. The interaction between colloidal particles was also evaluated using zeta potential analysis, and the values recorded ranged from −12.43 (5-FU-NE) to 2.11 mV (5-FU-C-NE). The presence of anionic groups of fatty acids and glycols in surfactants and co-surfactants results in the negative charge of the nanoemulsion (5-FU-NE). Chitosan possesses a positive charge and it was shown from the study that the addition of chitosan increases the zeta potential of the prepared nanoemulsion (−12.43 to 2.11) (*t*-test, *p* < 0.05).

**Figure 2:**
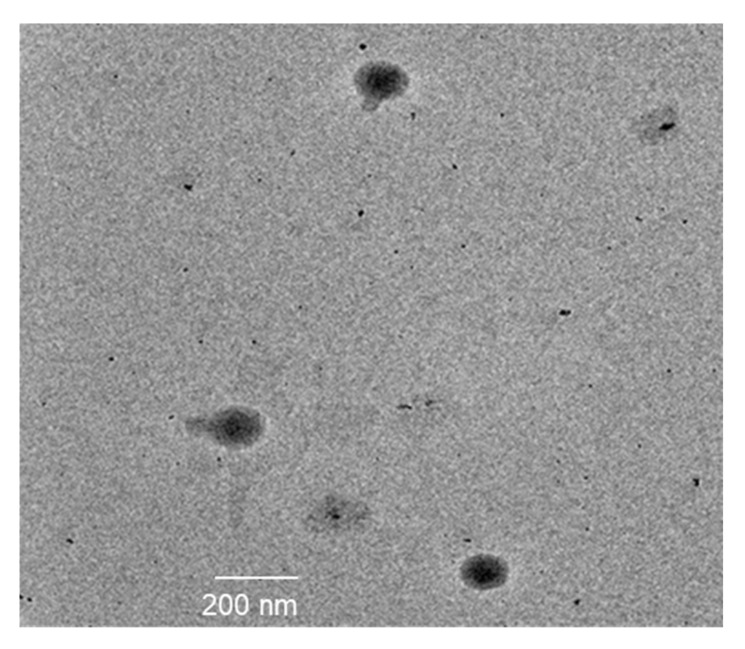
TEM image of nanoemulsion.

The surface morphology of the chitosan-coated nanoemulsion (5-FU-C-NE) was examined using TEM (Figure 2). It was observed from the TEM images of 5-FU-C-NE that particles were uniformly distributed. The shape of the globules was spherical and non-aggregated, indicating the improved stability of the formulation.

The pH of the formulated nanoemulsion ranged from 5.4 to 5.8 (Table 1). The addition of chitosan results in slightly lower pH. It might be due to the acidic nature of chitosan. The pH of the formulated nanoemulsion is compatible with the skin pH range.

#### 2.2.2. Entrapment Efficiency

The entrapment efficiency of 5-FU ranged between 51.2 ± 2.13 to 74.1 ± 2.41%. Nanoemulsion (5-FU-C-NE, 74.1 ± 2.41%) exhibited higher entrapment efficiency due to the presence of chitosan (*t*-test, *p* < 0.05) as compared to 5-FU-NE. The entrapment efficiency of the formulated nanoemulsion increases in the presence of chitosan. Mixed vesicles and micelles are present at higher surfactant concentrations, resulting in low drug entrapment in the mixed micelles [22].

#### 2.2.3. Stability

The nanoemulsion exhibited a good thermodynamic stability profile. No significant changes were observed in pH, physical appearance, zeta potential and particle size under extreme conditions (Table 2). At extreme conditions (40 °C), the optimized formulation 5-FU-C-NE exhibited the best and most satisfactory result and showed no significant changes in physicochemical parameters.

### 2.3. Physicochemical Characterization of Nanoemulsion Gel

#### 2.3.1. pH and Viscosity of Nanoemulsion Gel

The pH of the prepared nanoemulsion gels was in the range of 6.01 to 6.13 (Table 3). This pH is within the normal pH range of skin and would not produce any skin irritation, as confirmed by skin irritation studies. The presence of chitosan slightly reduces the pH of the gel. The viscosity of the topical formulation also plays an important role. The higher the viscosity of the topical formulation, the lower the drug release. The viscosity of nanoemulsion gel was higher than the simple gel (ANOVA; *p* < 0.05). This might be due to chitosan and oleic acid in the nanoemulsion gel. The viscosity of formulated 5-FU-C-NE-G was 12380 cps, which is considered the optimum viscosity for topical formulations. The optimum viscosity of topical formulations helps them to remain on the skin for a longer period.

#### 2.3.2. Spreadability and Drug Content

The spreadability of the formulated nanoemulsion gel was in the range of 5.8 to 6.4 g cm/s (Table 3). These values are indicative of the good spreadability of the nanoemulsion gels. The spreadability of nanoemulsion gel was slightly higher than the simple gel due to the presence of chitosan. The spreadability values indicate that the formulated nanoemulsion gel is easily spreadable by applying just a small force. The drug content of the prepared gels was in the range of 91.32% to 92.41%, within the acceptable range of 85–110%.

### 2.4. In Vitro Release Studies

**Figure 3:**
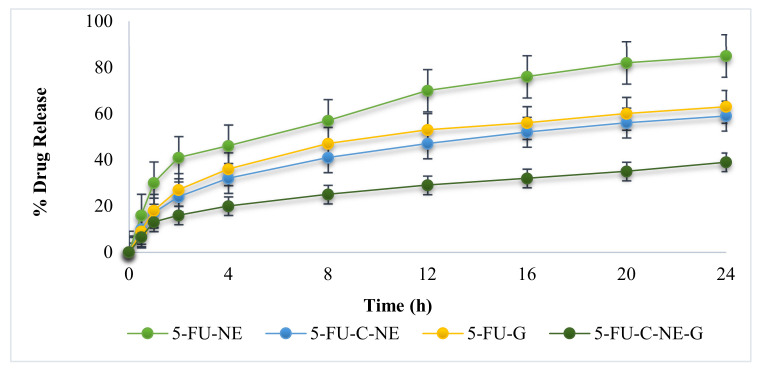
Profile of in vitro drug release.

Initially, the burst release of 5-FU occurred from the nanoemulsion formulation due to the 5-FU outer layer eroded from 5-FU-NE and 5-FU-C-NE. The burst release was followed by the sustained release as shown in Figure 3. The 5-FU-NE and 5-FU-G formulations exhibited a higher release of 5-FU (ANOVA, *p* < 0.05). The addition of chitosan to the nanoemulsion reduced the release of 5-FU. Chitosan is a cationic, biodegradable polymer used as a controlling rate agent in drug delivery. The results revealed that the coating layer of chitosan was an effective barrier to 5-FU release. Drug release from chitosan polymers depends on hydrophilicity, degree of swelling and molecular weight. The incorporation of 5-FU-C-NE into gel further reduces the release of 5-FU. 5-FU eroded from the outer layer of the nanoemulsion and nanoemulsion gel, resulting in the initial burst of 5-FU. The mechanism of drug release was evaluated through the Korsmeyer–Peppas model. Values of n < 0.45 indicates Fickian diffusion and values of n > 0.45 indicate non-Fickian diffusion [16]. The value of “n” indicates the linear regression.

It was found that both formulated nanoemulsion and nanoemulsion gel fitted the Korsmeyer–Peppas model and the release exponent (n) values calculated for the 5-FU-C-NE and 5-FU-C-NE-G were 0.672 and 0.735, respectively. The study depicted that the prepared formulations followed non-Fickian diffusion.

### 2.5. Ex Vivo Permeation

**Figure 4:**
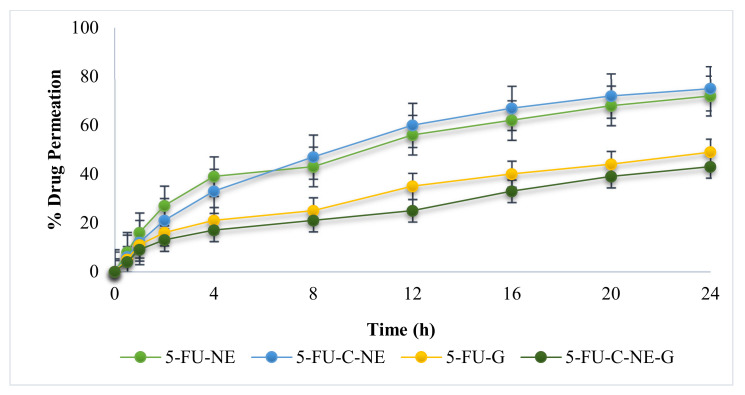
Profile of ex vivo drug permeation.

The ex vivo permeation results of the formulated nanoemulsion and gel are shown in Figure 4. The permeation of 5-FU was higher in the case of 5-FU-C-NE compared to 5-FU-NE (ANOVA, *p* < 0.05). The chitosan decoration helps to increase the permeation of 5-FU. Chitosan acts as a permeation enhancer by reversibly fluidizing the lipids and proteins (keratin and ceramides) [23]. Similar results were found by Rehan et al., 2022, where the chitosan decoration of nanoemulsion helped the skin drug delivery [13]. Using nanoemulsion reduces the barrier nature of the stratum corneum because it acts as a permeation enhancer. The ex vivo permeation study revealed that nanoemulsion exhibited greater permeation compared to the gel formulation (5-FU-G and 5-FU-C-NE-G) (ANOVA, *p* < 0.05) (Figure 4). The reason might be that the nanoemulsions are more viscous than gels and have a low ability to penetrate the skin barrier layer (Figure 4). In addition, nanoemulsions have a small droplet diameter and high drug concentration, and the reaction between skin stratum corneum and surfactants enables greater permeation than nanoemulsion gel formulations. In previous studies, nanoemulsion gels of 5-FU were prepared for the topical delivery [20]. The percentage of drug release and permeation were higher for the nanoemulsion gel of 5-FU as compared to the 5-FU solution due to the synergistic effect of nanoemulsion and excipients used. Furthermore, it was found that the % permeation of 5-FU was higher in the case of 5-FU-C-NE-G as compared to its release. This indicates that some of the 5-FU permeated in an intact form without the release/rupture of droplets.

### 2.6. Skin Drug Retention

**Figure 5:**
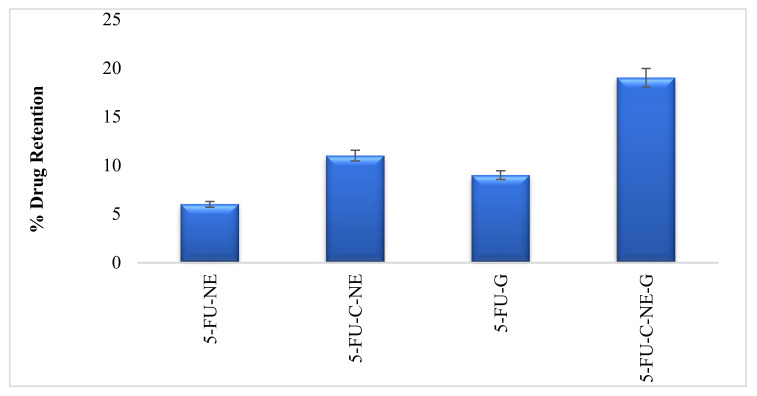
Skin drug retention.

The retention of 5-FU in the skin peaked when 5-FU-C-NE-G was used (Figure 5). Minimum skin drug retention was found in the case of the simple nanoemulsion (5-FU-NE). Incorporating chitosan has significantly increased skin drug retention (ANOVA, *p* < 0.05). This is due to the positive charge of chitosan, which interacts with the negatively charged skin, resulting in higher skin drug retention.

The presence of chitosan and Carbopol resulted in the significantly higher deposition of 5-FU from the prepared formulation (5-FU-C-NE-G) (ANOVA, *p* < 0.05). Carbopol is a cross-linked polymer that can form a matrix reservoir. The reservoir slowly releases the drug over an extended period of time [20]. The Carbopol gel offered entrapment of lipid vesicles and showed an intra-matrix system that results in the accumulation of a core amount of 5-FU in the deeper layers of the skin. The maximum amount of 5-FU retention in the deeper layer of the skin was observed with the prepared formulation (5-FU-C-NE-G). Hence, the controlled and maximum skin retention was achieved by the prepared formulation (5-FU-C-NE-G) with the lowest systemic absorption of 5-FU. A higher concentration of 5-FU in the deeper layers of skin helps treat skin cancers such as melanoma, which originates from a deeper layer of skin.

### 2.7. Skin Irritation

The prepared formulations were subjected to the skin irritancy test. The prepared formulations were compared with the standard formalin, and the study showed that all prepared formulations resulted in no skin irritation compared to the standard irritant (formalin). The formulated nanoemulsion gel caused no skin irritation, indicating it is safe to be used on the skin. The skin irritation study showed that the prepared nanoemulsion gel is safe for skin application and the topical drug delivery of 5-FU.

### 2.8. In Vivo Studies

**Figure 6:**
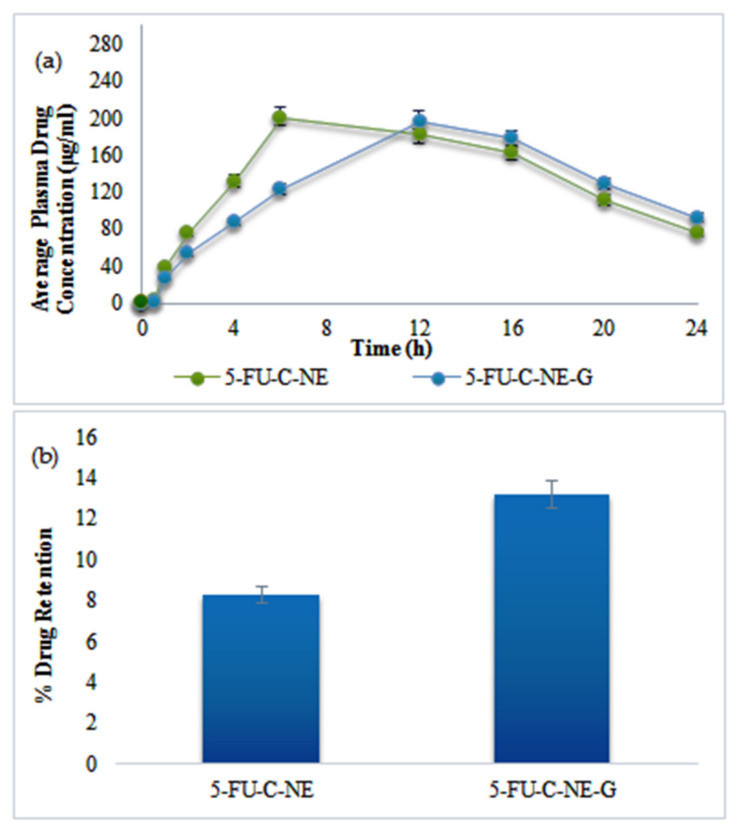
Average plasma drug concentration in µg/mL (**a**) and skin drug retention (**b**).

Maximum plasma concentration was observed for 5-FU-C-NE with s C_max_ of 201.3 µg/mL within 6 h of administration (Figure 6a). The area under the curve (AUC) values were comparable in both cases. C_max_ of 5-FU-C-NE-G at 199.4 µg/mL was achieved after 8 h, possibly due to slower drug release from the gel matrix. The half-life of 5-FU-C-NE-G (9 h) was significantly higher than that of 5-FU-C-NE (12 h). The percentage of skin drug retention was significantly higher in the case of 5-FU-C-NE-G compared to nanoemulsion (ANOVA: *p* < 0.05). A reduction in the permeation tendency of the drug into systemic circulation enables increased skin drug retention in rats treated with 5-FU-C-NE-G. The result indicates that rats treated with nanoemulsion gel were favorable for local skin diseases such as melanoma (Figure 6b).

## 3. Conclusions

Nanoemulsion and nanoemulsion incorporated gel were successfully prepared for the topical delivery of 5-FU. The physicochemical characterization of the prepared nanoemulsion and gel, in vitro release, ex vivo permeation and in vivo profiles were studied. The results showed that adding chitosan controlled the release of 5-FU and increased skin drug retention. The incorporation of chitosan-coated nanoemulsion into gel further reduced the release and enhanced the skin drug retention compared to nanoemulsion and simple gel. The optimized formulation (5-FU-C-NE-G) could retain 5-FU in the skin for a prolonged period and was proven a non-irritant. The results of in vivo studies also confirm that 5-FU-C-NE-G is associated with higher skin retention of the drug, which is suitable for treating local skin diseases such as melanoma.

## 4. Materials and Methods

### 4.1. Materials

5-FU was purchased from Sigma-Aldrich, USA (99% pure). Oleic acid (Sigma-Aldrich, St. Louis, MO, USA), was used in the preparation of the oil phase. Tween 20 and glycerol (The Dow Chemical Company., 693 Washington St. #627, Midland, MI 48640, USA), were used as surfactant and co-surfactant, respectively. Chitosan (degree of deacetylation (DD) 83% and molecular weight 310,000–375,000) was used as a coating agent to control the release of 5-FU and Carbopol was used as a gelling agent (Sigma-Aldrich, St. Louis, MO, USA).

### 4.2. Preparation of Nanoemulsion

The aqueous phase was prepared by dissolving 1% (*w*/*w*) of 5-FU in distilled water and the oil phase was prepared with oleic acid (20% *w*/*w*) (Table 4). Tween 20 (8% *w*/*w*) and glycerol (2% *w*/*w*) were used as surfactant and co-surfactant, respectively. Pre-emulsion was prepared by drop-wise introduction of the aqueous phase to the oil phase with continuous stirring at 25 °C. High-shear homogenization was carried out at 10,000 rpm for a time period of 10 min.

### 4.3. Compatibility Studies

The interaction of 5-FU with excipients was evaluated by ATR-FTIR (PerkinElmer, Washington, DC, USA). The pure drug (5-FU), chitosan, excipients, nanoemulsion and gel spectra were observed via an attenuated total reflectance (4000–400 cm^−1^). All samples were analyzed without any particular preparations [24].

### 4.4. Physicochemical Characterization of Nanoemulsion

#### 4.4.1. Size and Morphology

The particle size of the nanoemulsion was evaluated using Dynamic Light Scattering (Malvern, UK). One milliliter of nanoemulsion was diluted with nine milliliters of distilled water and analyzed using a Zetasizer. Data were expressed as mean ± SD [25]. A transmission electron microscope (TEM CMIZ, Philips EM400, Kyoto, Japan) was used to examine nanoemulsion morphology. One drop of nanoemulsion was kept on the grid and visualized by TEM at 80 KV [26].

#### 4.4.2. Entrapment Efficiency

Centrifugation of the prepared nanoemulsion was carried out at 1000 rpm for 2 h at 25 °C. The supernatant containing free drug and pellets (containing entrapped drug) of nanoemulsion was obtained. The obtained pellets were dissolved in acetic acid solution (1% *v*/*v*) to release the entrapped drug. UV Spectrophotometer (Shimadzu UV 1801, Kyoto, Japan) was utilized to evaluate the drug concentration of supernatant and dissolved pellets. The following formula was used for evaluating entrapment efficiency:

Entrapment efficiency = (A − B/A) × 100 (1)

where A is the initial amount of 5-FU, B is the 5-FU amount in the filtrate, and (A − B) represents the entrapped amount of 5-FU in the formulation [27].

### 4.5. Preparation of Nanoemulsion Gel

The optimized nanoemulsion was incorporated in the polymer (Carbopol 940) (The Dow Chemical Company., 693 Washington St. #627, Midland, MI, USA), dispersion to form nanoemulsion gel. In a beaker, 100 mL of distilled water was taken and 1 g of Carbopol 940 was added and allowed to disperse completely. After complete dispersion, triethanolamine was incorporated dropwise until a clear gel formulation was prepared. The nanoemulsion and gel base was mixed in a 1:1 ratio with constant stirring. The prepared nanoemulsion was observed for different parameters.

### 4.6. Physicochemical Characterization of Nanoemulsion Gel

#### 4.6.1. pH and Viscosity of Nanoemulsion Gel

A digital pH meter (Sartorius Mechatronics Japan KK, Kyoto, Japan) was used to measure the pH of the prepared nanoemulsion gel. The data were taken in triplicate and expressed as mean ± SD [28].

Brookfield viscometer (Brookfield Engineering Laboratories, Inc., Middleboro, MA, USA) was used to measure the viscosity of the prepared nanoemulsion gel. The data were conducted in triplicate and expressed as mean ± SD [29].

#### 4.6.2. Spreadability

The spreadability of the prepared nanoemulsion gel was measured using modified apparatus comprising of two glass slides with the sample placed in between these slides. The upper slide was connected to a balance by a hook and the lower slide was fixed to the wooden plate. The following formula was used for evaluating the spreadability of the prepared nanoemulsion gel:

S = M × l/t (2)

where S represents the spreadability (g cm/s), M represents the mass of gel deposited in between these two slides, l represents the length of slide and t is the detachment time of upper slide [30].

#### 4.6.3. Drug Content

For the evaluation of drug content, 1 g of nanoemulsion was mixed with 99 mL of phosphate buffer solution at pH 7.4 for 24 h with continuous stirring on a magnetic stirrer. The clear solution obtained was analyzed at λmax 265 nm via UV spectrophotometer [31].

### 4.7. In Vitro Release Studies

In vitro drug release experiment was conducted using a Franz diffusion cell (Perme-Gear, Leithsville Rd, Hellertown, PA, USA). This study was performed to evaluate in vitro drug release behavior of the drug. One milliliter of nanoemulsion was placed on the donor compartment’s Tuffryn membrane (artificial membrane, pore size 0.45 µm). Phosphate buffer at pH 5.5 (to simulate skin pH) was used to fill the receptor compartments and the temperature was maintained at 32 ± 0.5 °C (to simulate skin temperature). At specific time intervals (0.5, 1, 1.5, 2, 4, 8, 12, 16 and 24 h), aliquots of 2 mL were obtained from the receptor compartment and compensated with fresh phosphate buffer solution (pH 5.5) to maintain the sink conditions. UV spectrophotometry at 265 nm wavelength was employed to analyze the collected aliquots [32].

#### Release Kinetics

Zero-order, first-order and Higuchi kinetic models were applied to the collected samples to examine the formulated system’s drug release profiles. Korsmeyer–Peppas equation was also used to investigate the mechanism of drug release and the exponent was quantified from the slope of a straight line.

### 4.8. Ex Vivo Drug Permeation Studies

Franz diffusion cell was utilized for evaluating ex vivo permeation profiles across the skin of male Sprague Dawley rats. The rat handling procedure was approved by the Ethics Review Board, Gomal University, Pakistan (204/QEC/GU/2020). The animal was sacrificed with an overdose of ketamine/xylazine injection. The dorsal region of the rat was removed with the help of scissors and a surgical blade. The excised skin was separated and adhered fats were removed surgically. Then, the excised skin was rinsed with normal saline 0.9%. The excised skin was placed over the receptor compartment (0.77 cm^2^) so that the dermal part of the skin faced the receiving compartment and the stratum corneum side faced the donor compartment. The receptor compartments were filled with a medium consisting of phosphate buffer solution pH 7.4 (to simulate blood pH) and 1% sodium azide (to prevent microbial growth). The temperature was maintained at 37 ± 1.0 °C (to simulate blood temperature). The prepared membrane was applied to the region of the donor compartment. At predetermined time intervals of 0, 0.5, 1, 2, 4, 8, 12, 16, 20 and 24 h, the required aliquot volumes (2 mL) were obtained and fresh phosphate buffer solution (pH 7.4) was added to maintain the sink condition [33]. The samples were analyzed on a UV spectrophotometer at 265 nm wavelength.

### 4.9. Skin Drug Retention Studies

The amount of 5-FU retained in the deeper layers of the skin was evaluated after the completion of ex vivo studies. The skin was collected, cut into small pieces and washed with saline water from the Franz diffusion cells. Five milliliters of methanol were utilized for the homogenization of the skin sample. The solution was centrifuged for 5 min at 5000 rpm and the drug was quantified by UV spectrophotometry at 265 nm wavelength [34]

### 4.10. Skin Irritation Studies

Sprague Dawley rats were used for the evaluation of skin irritation studies. The rats were anesthetized using ketamine/xylazine solution. The skin of the rats was shaved with a sharp razor. 5-FU-C-NE-G and formalin (standard irritant) were applied twice daily to the shaved area. The readings were taken for up to 12 h [29].

### 4.11. In Vivo Studies

This study used male Sprague Dawley rats (200–250 g each). The chitosan-coated nanoemulsion (5-FU-C-NE) and nanoemulsion gel (5-FU-C-NE-G) were used in in vivo studies. In total, 12 rats were divided into two groups (A and B, n = 6). The study protocol was approved by the Ethical Review Board, Gomal University, Pakistan. The rats were anesthetized and the dorsal region was shaved with sharp blades. One gram each of 5-FU-C-NE (group A) and 5-FU-C-NE-G (group B) was applied to the shaved area of the rat. Then, 0.5 mL of blood was sampled at specific intervals (0, 0.5, 2, 4, 8, 16 and 24). The plasma drug concentrations were quantified using high-performance liquid chromatography (HPLC) [35]. Pharmacokinetic parameters were calculated using kinetica 5.0 software. After 24 h, the rats were sacrificed via cervical dislocation and the treated skin area was removed. The skin was cut into pieces and the drug was extracted using 5 mL methanol. HPLC was used for the evaluation of skin drug retention.

### 4.12. Statistical Analysis

The experiments were performed in triplicate, calculated and averaged as mean ± SD. One-way ANOVA statistical test followed by a post hoc test (Tukey’s honestly significant difference test) was used. In vivo studies were analyzed via two-way ANOVA. Statistical analysis was performed using SPSS version 16 software (IBM, Chicago, IL, USA) and *p* < 0.05 was considered statistically significant.

## Figures and Tables

**Table 1:** Physicochemical properties of the nanoemulsion.

F. Code	Size (nm)	Zeta Potential	PDI	pH	%EE
5-FU-NE	92.14 ± 2.35	−12.43 ± 1.67	0.23 ± 0.04	5.81 ± 0.67	51.23 ± 2.13
5-FU-C-NE	119.32 ± 2.87	2.11 ± 1.24	0.25 ± 0.12	5.43 ± 0.93	74.14 ± 2.41

**Table 2:** Physicochemical properties of the formulated nanoemulsion at 40 °C.

F. Code	Size (nm)	Zeta Potential	PDI	pH	%EE
5-FU-NE	94.43 ± 2.67	−10.99 ± 1.83	0.26 ± 0.13	5.73 ± 0.81	50.13 ± 2.56
5-FU-C-NE	123.12 ± 2.35	2.03 ± 1.15	0.28 ± 0.17	5.37 ± 0.89	72.42 ± 2.53

**Table 3:** Physicochemical characterization of the nanoemulsion gels.

F. Code	pH	Viscosity (cps)	Spreadability (g cm/s)	Drug Content (%)
5-FU-G	6.13 ± 1.04	11,678 ± 10.94	5.8 ± 1.32	92.41 ± 2.31
5-FU-C-NE-G	6.01 ± 1.53	12,380 ± 11.72	6.4 ± 1.89	91.32 ± 2.82

**Table 4:** Composition of the nanoemulsion.

F. Code	Drug (%)	Chitosan (%)	Oleic Acid (%)	Tween 20 (%)	Glycerol (%)
5-FU-NE	1	-	20	8	2
5-FU-C-NE	1	0.25	20	8	2

## Data Availability

Not applicable.
